# Influence of cochlear coverage on speech perception in single sided deafness, bimodal, and bilateral implanted cochlear implant patients

**DOI:** 10.1007/s00405-024-09086-x

**Published:** 2024-12-17

**Authors:** Jennifer L. Spiegel, Joachim Mueller, Rebecca Boehnlein, John-Martin Hempel, Judith E. Spiro, Bernhard G. Weiss, Mattis Bertlich, Martin Canis, Tobias Rader

**Affiliations:** 1 Department for Otorhinolaryngology, University Hospital, Ludwig-Maximilians-Universität München, Marchioninistr. 15, 81377 Munich, Germany; 2 Department of Radiology, University Hospital, Ludwig-Maximilians-Universität München, Marchioninistr. 15, 81377 Munich, Germany

**Keywords:** Cochlear coverage, Speech perception, SSD, Single sided deafness, Bimodal

## Abstract

**Purpose:**

Individualized cochlear implantation (CI) is essential to facilitate optimal hearing results for patients. Influence of cochlear coverage (CC) has been studied, however without consideration of different CI-categories, like single sided deafness (SSD), bimodal, and bilateral separately.

**Methods:**

Retrospective analysis of preoperative CT scans was performed at a tertiary center. For each patient their individual CC with the selected electrode array was calculated off the complete CDL. Patients were categorized into SSD (n = 30), bimodal (n = 72), and bilateral CI patients (n = 29). Speech perception within the first 12 months post-implantation was compared between patient groups with shorter and longer CC. For subgroup analysis the cutoff between a shorter or longer CC was identified by the median.

**Results:**

Cutoff between a shorter or longer CC was identified at 65% off the complete CDL for SSD and bimodal patients, and at 70% for bilateral patients. In SSD-patients longer CC was associated with better performance at activation (CC^shorter^ 20.0 ± 28.9% vs. CC^longer^ 31.5 ± 24.7%; *p* = 0.04) and no benefit was found with deeper insertion at 12 months. No significant benefit was found for deeper insertion in bimodal and bilateral patients.

**Conclusions:**

Capacities of hearing performance seem to differ between SSD, bimodal and bilateral patients within the first year after implantation with regards to cochlear coverage. SSD-patients appear to benefit from deeper insertion than 65% up to 12 months after implantation. However, these results should be interpreted with caution, hence development of speech perception with CI is influenced by a whole range of factors, and bimodal and bilateral treated patients are extremely heterogenous patient groups.

## Introduction

Hearing loss is a major global health concern affecting 1.5 billion people with rising numbers expected due to an aging population [1]. For patients suffering from deafness, a cochlear implant (CI) can help to restore hearing. Essentially, patients with CI can be divided into five different categories: (1) single sided deafness (SSD): patients with one deaf and one normal hearing ear [2]. (2) asymmetric hearing: hearing aid (HA) on the better ear without CI indication and CI on the worse ear [2]. (3) bimodal: patients who meet the requirements for CI for both ears but have received a CI on one ear and still use a HA on the other [3]. (4) bilateral: patients with CIs on both ears [4]. (5) electroacoustic stimulation: patients with normal to mildly impaired low-frequency residual hearing and down sloping high frequency deafness, who receive an into the CI audio processor integrated HA to allow simultaneous acoustic and electrical stimulation [5, 6]. Hearing results for all those different categories should be interpreted separately since the hearing learning process with the CI varies for all of them. Therefore, individualized cochlear implantation is essential to facilitate optimal hearing results for patients. This includes considering the diverse anatomical characteristics of each patient, such as inner ear malformations [7, 8] and a wide ranging length of the cochlea [9–11]. Different CI manufacturers offer a variety of electrodes with different lengths and locations within the cochlea [12–15]. Measuring the cochlear duct length (CDL) and thus estimating the cochlear coverage (CC) or the angular insertion depth (AID) has been simplified with software that automatically calculate those values on a computed tomography (CT) scan of the temporal bone [16–22]. Hence, anatomy-based fitting by applying the Greenwood function [23, 24] has become a novel approach for fitting the CI [25–29]. Groups have been studying the influence of cochlear coverage on speech perception of CI patients [30–35]. In all those studies no differentiation with regards to the CI categories SSD, bimodal or bilateral was made. Therefore, the current study investigated the influence of cochlear coverage on the speech perception within the first year after CI for the different CI categories SSD, bimodal or bilateral separately.

## Material and methods

### Patient selection and ethical considerations

**Fig. 1:**
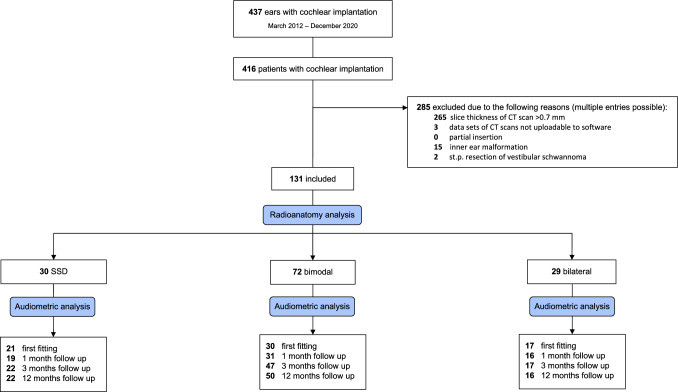
Consolidated Standard Reporting of Trials (CONSORT) Flow Diagram

The study is a retrospective analysis of 131 patients´ preoperative CT images of the temporal bone. Patients received cochlear implantation with x-ray confirmed full insertion of either FLEX28, FLEXSOFT or STANDARD electrode of the company MED EL (MED EL GmbH, Innsbruck, Austria) between March 2012 and December 2020. Out of a total of 423 patients, 131 CT scans (radiologic *Digital Imaging and Communications in Medicine*, DICOM®) with a thickness < 0.7 mm were found eligible for analysis with the OTOPLAN software. Exclusion criteria were revision implantation, a slice thickness of the CT scan > 0.7 mm, cochlear malformations, partial insertion as suggested by postoperative stenvers-x-ray, status post vestibular schwannoma resection, and data sets which could not be uploaded to the software. Postoperative stenvers x-ray confirmed full insertion and no tip foldover in all of the included patients (see CONSORT flow diagram, Fig. 1).

The patients were then sorted in three different groups: those with single sided deafness (SSD), bimodal, and bilateral implantation. SSD was defined as patients with ipsilateral ear with criteria for cochlear implantation and contralateral ear with normal hearing to mild sensorineural hearing loss without indication for hearing aid. Bimodal was defined as patients with ipsilateral ear with indication for cochlear implantation and contralateral ear with hearing aid and borderline indication for cochlear implant. Bilateral patients had received cochlear implants on both ears. The study was approved by the local ethics committee. Procedures were followed in accordance with ethical standards of the Helsinki Declaration [36].

### Software and data analysis

The preoperative images were uploaded into OTOPLAN version 3.0 (CE-certification number: G1 17 10 95,657 003), which was developed by CAScination AG (Bern, Switzerland) [37]. With OTOPLAN the cochlear duct length (CDL) was measured by two double-blinded (blinded to the electrode and the other rater’s results) raters independently using multiplanar reformation. The measurements were performed as described before [11]. In short, the plane depicting the whole basal turn was reconstructed to measure first ‘A-value’, defined as the largest distance from the round window to the contralateral wall, and then ‘B-value’, defined as the distance between cochlear walls perpendicular to the ‘A-value’-line. Then, on an orthogonal plan ‘H-value’ as the height of the cochlear was determined. The software then calculates the cochlear duct length according to the elliptic-circular approximation (ECA) method, as well as angular insertion depth (AID), the cochlear coverage (CC) and cochlear place frequency on the basis of the Greenwood function with respect to the selected electrode.

The CC was calculated off the complete CDL as the coverage of the cochlear by the selected electrode array individually for each patient. Therefore, a cochlear coverage of 100% means full coverage of the complete CDL. For example, if a patient with a CDL of 35 mm would receive a 28 mm electrode array, this would result in a CC of 80%. Whereas in a patient with a CDL of 41 mm, who receives a 28 mm electrode array, this would result in a CC of 68.3%. Therefore, we selected the CC as the parameter to evaluate for shorter and longer coverage.

After determining the CC for each implanted ear, patients were assigned to their CI category: SSC, bimodal and bilateral. For each group, the median of the CC was calculated: SSD = 65%; bimodal = 65%; bilateral = 70%. Subgroup analyses were done in accordance with this determined cutoff (= median): all patients below the calculated median were assigned to the subgroup “shorter CDL”, all above to the subgroup “longer CDL”.

### Fitting

All patients received the activation of their cochlear implant 4–6 weeks postoperatively and fitting at 1, 3, 6, 9, and 12 months after activation [38].

### Speech audiometry

Evaluation of speech discrimination of monosyllabic words in quiet was performed with the German language Freiburg Monosyllabic Test [39–41], at time point of first fitting and re-fittings of the cochlear implant. During the test, 20 previously recorded monosyllabic words from a male speaker were presented to the patient in quiet at various sound levels to achieve the individual sound presentation level with best speech perception (= dB opt). Word recognition score (WRS) was measured in quiet at 65 dB hearing level (HL) with the contralateral ear plugged. Each correctly recognized word was accounted for 5%, the score for normal hearing subjects is 100%. Speech audiometry was not obtained consistently, thus, only the subset is reported.

### Pure tone audiometry

Pure tone audiometry was performed for each ear at the frequencies 0.125, 0.25, 0.5, 0.75, 1, 1.5, 2, 3, 4, 6, and 8 kHz via headphone and with air and bone conduction for each ear separately. Aided air conduction was measured with warble tones in free field. Thresholds exceeding 120 dB HL were recorded as 120 dB HL for statistical purposes. The audiometry was performed at timepoints of first fitting and re-fittings of the device.

The pure tone average at the frequencies 0.5, 1, 2, 4 kHz (PTA4) and for low frequency hearing at 0.125, 0.25 Hz and 0.5 Hz (PTA^low^) was calculated to determine if and how much hearing of lower tones was still possible for patients preoperatively. This is seen as an indicator for functional usage of acoustic stimulation [42].

### Statistical analysis

For the statistical analysis and the creation of tables and figures, the software Statistical Package for Social Sciences (SPSS) Software (IMB, Armonk, NY, USA, Version 29) and Microsoft Excel (Microsoft, Redmond, WA, USA, Version) were used. Shapiro-Walk test was used to test for normative distribution. Due to the majority of not normally distributed data, a non-parametric test (Mann–Whitney-U-Test) was utilized for comparison analysis.

## Results

### Patients’ characteristics and cochlear coverage

**Fig. 2:**
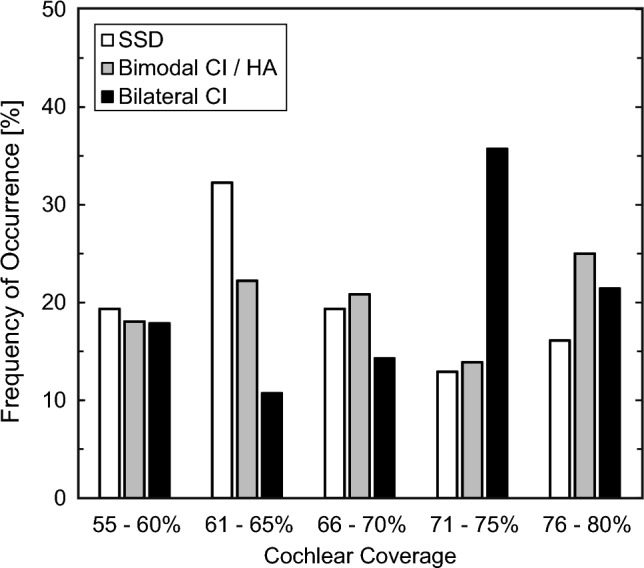
Distribution of Cochlear Coverage (CC) for the Patient Groups Single Sided Deafness (SSD), Bimodal, and Bilateral

In this retrospective study 131 patients´ cochleae with x-ray confirmed full insertion were measured using the OTOPLAN software (Fig. 1). 66 of these patients were male (49.6%). The patients were sorted into three groups: SSD (n = 30), bimodal hearing (n = 72), and bilateral implanted (n = 29) of which all received a cochlear implant by the company MED EL. As shown in Table 1, in the SSD group, the mean age was 45.7 ± 16.1 years and 46.7% of the measured ears were right ears. The majority of the SSD-patients (81.3%) had sudden hearing loss (idiopathic sudden sensorineural hearing loss, hearing loss after otologic surgery) with a short time span (3 months–5 years) from onset of hearing loss until receiving the CI. 56.7% received a FLEX28, 20% received a FLEXSOFT, and 23.3% received a STANDARD electrode array. In the group with bimodal hearing the mean age was 61.9 ± 17.8 years and 56.9% of the measured ears were right ears. 66.7% of the patients received a FLEX28, 33.3% received a FLEXSOFT. In the bilateral implanted group 55.2% of the measured ears were the first ear to be implanted. The mean age of these patients was 43.2 ± 18.3 years and 51.7% of the measured ears were right ears. The patients received in 37.9% a FLEX28, in 51.7% a FLEXSOFT, and in 10.4% a STANDARD electrode array. The mean CDL in the whole cohort was 36.5 ± 1.8 mm with almost no variation between the three groups. The mean AID was 555.1 ± 60.6° in the SSD group, 557.1 ± 61.8° in the bimodal group, and 588.0 ± 67.1° in the bilateral group. The resulting mean CC in the SSD group was 66.1 ± 6.5%, 68.2 ± 7.5% for bimodal, and 69.9 ± 7.7% for bilateral patients. All demographic data is depicted in Table 1. Figure 2 displays the distribution of the cochlear coverage for all patient categories. Notable is the higher rate of patients with longer coverage in the bilateral group (Fig. 2).

**Table 1:** Patients’ characteristics and cochlear coverage

	CC < 65%	CC > 65%	Total
SSD [n (%)]	16 (53.3)	14 (46.7)	30 (100)
Female [n (%)]	6 (37.5)	7 (50.0)	13 (43.3)
Age [years ± SD]	45.4 ± 16.3	45.9 ± 16.4	45.7 ± 16.1
SSNHL [n (%)]	13 (81.3)	13 (81.3)	26 (81.3)
Side (right) [n (%)]	9 (56.3)	5 (35.7)	14 (46.7)
Electrode array			
Flex28 [n (%)]	14 (87.5)	3 (21.4)	17 (56.7)
FlexSoft [n (%)]	1 (6.25)	5 (35.7)	6 (20.0)
Standard [n (%)]	1 (6.25)	6 (42.8)	7 (23.3)
CDL [mm ± SD]	37.7 ± 1.4	36.6 ± 2.0	37.1 ± 1.8
AID [° ± SD]	508.9 ± 23.7	607.9 ± 43.6	555.1 ± 60.6
CC [% ± SD]	61.2 ± 2.5	71.8 ± 4.7	66.1 ± 6.5
A-value [mm ± SD]	9.8 ± 0.5	9.2 ± 0.7	9.5 ± 0.7
B-value [mm ± SD]	7.2 ± 0.2	7.1 ± 0.4	7.2 ± 0.3
Bimodal [n (%)]	29 (40.3)	43 (59.7)	72 (100)
Female [n (%)]	13 (44.8)	23 (53.5)	36 (50.0)
Age [years ± SD]	60.9 ± 19.1	62.6 ± 17.0	61.9 ± 17.8
Side (right) [n (%)]	17 (58.6%)	24 (55.8)	41 (56.9)
Electrode array			
Flex28 [n (%)]	29 (100)	19 (44.2)	48 (66.7)
FlexSoft [n (%)]	0 (0)	24 (55.8)	24 (33.3)
CDL [mm ± SD]	37.2 ± 1.2	35.3 ± 1.8	36.0 ± 1.8
AID [° ± SD]	500.0 ± 25.3	595.5 ± 47.6	557.1 ± 61.8
CC [% ± SD]	60.9 ± 3.1	73.2 ± 5.1	68.2 ± 7.5
A-value [mm]	9.5 ± 0.3	9.2 ± 0.4	9.4 ± 0.4
B-value [mm]	7.3 ± 0.3	6.8 ± 0.4	7.0 ± 0.4

	CC < 70%	CC > 70%	Total
Bilateral [n (%)]	12 (41.4)	17 (58.6)	29 (100)
Female [n (%)]	8 (66.7)	8 (47.1)	16 (55.2)
Age [years ± SD]	40.2 ± 20.1	45.3 ± 17.2	43.2 ± 18.3
Side (right) [n (%)]	6 (50.0)	9 (52.9)	15 (51.7)
Electrode array			
Flex28 [n (%)]	9 (75)	2 (11.8)	11 (37.9)
FlexSoft [n (%)]	1 (8.3)	14 (82.4)	15 (51.7)
Standard [n (%)]	2 (16.7)	1 (5.8)	3 (10.3)
CDL [mm ± SD]	37.2 ± 1.5	35.7 ± 1.8	36.3 ± 1.8
AID [° ± SD]	525.5 ± 47.4	634.9 ± 31.1	588.0 ± 67.1
CC [% ± SD]	62.5 ± 4.6	75.6 ± 3.6	69.9 ± 7.7
A-value [mm ± SD]	9.4 ± 0.5	9.2 ± 0.5	9.3 ± 0.5
B-value [mm ± SD]	7.2 ± 0.3	6.9 ± 0.4	7.0 ± 0.4
First implanted [n (%)]	8 (66.7%)	8 (47.1)	16 (55.2)
Second implanted [n (%)]	4 (33.3%)	9 (52.9)	13 (44.8)

Demographics and data on the received electrode array, as well as OTOPLAN measurements are given for all three patient groups single sided deafness (SSD), bimodal, and bilateral, as well as values for shorter and longer cochlear coverage (CC). For subgroup analysis the median cochlear coverage was identified at 65% for SSD and bimodal hearing patients, at 70% for bilateral patients

*AID* angular insertion depth, *CDL* cochlear duct length, *n* number of patients, *SD* standard deviation, *SSNHL* sudden sensorineural hearing loss

### Audiometric results with regards to the cochlear coverage

**Fig. 3:**
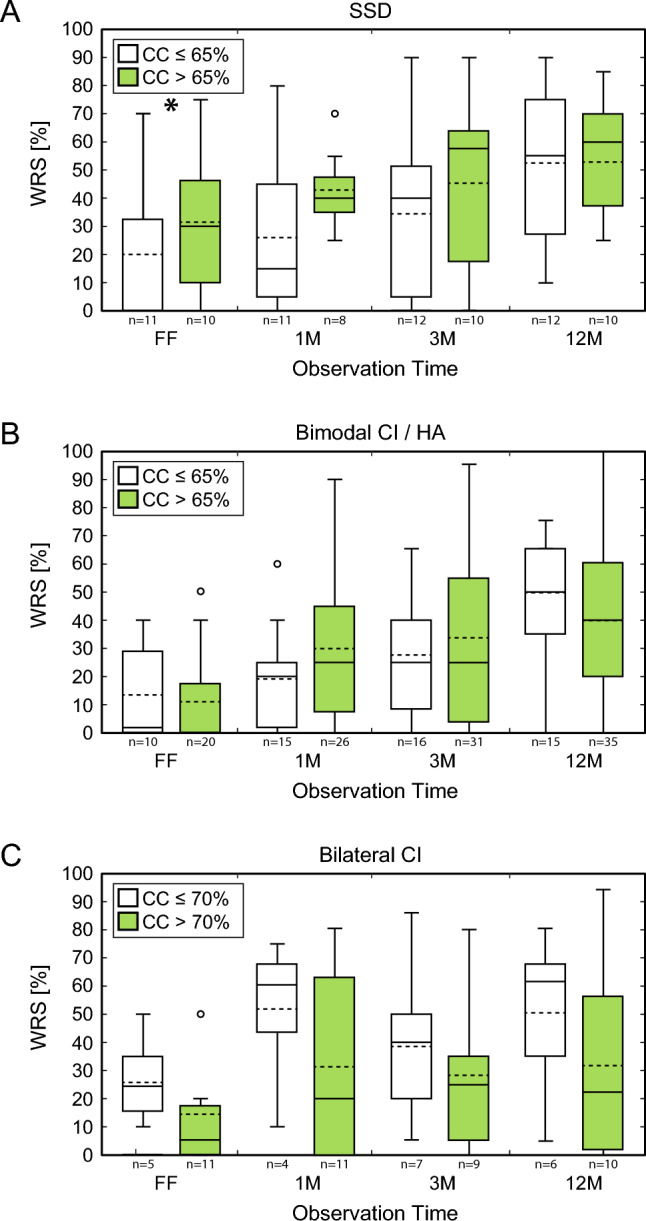
Speech Perception with Freiburg Monosyllabic Test with respect to the Cochlear Coverage for the Different Groups. For subgroup analysis the median cochlear coverage was identified at 65% for SSD and bimodal hearing patients, at 70% for bilateral patients. Data at first fitting (FF), 1 month (1 M), 3 months (3 M), and 12 months (12 M) are depicted in boxplots at the x-axis, the y-axis represents the correct identified monosyllables in percent of the German language Freiburg Monosyllabic Test [39–41]. White boxplots resemble shorter CC, green boxplots longer CC. Outliner values are depicted as small circles. **A** Shows results for SSD patients, **B** for bimodal, and **C** for bilateral. *, significant* p*-value (*p* < 0.05)

In order to distinguish between patients with a long CC and short CC, two subgroups were generated using the median, which was identified at a cutoff of > / < 65% for SSD and bimodal patients, and at a cufoff of > / < 70% for bilateral implanted patients. No significant differences were found in all three groups with regards to the preoperative PTA4 and PTA^low^, as the indicator for residual hearing (see Table 2). Timeline between preoperative audiogram and implant surgeries was similar for all three categories (SSD mean 2.8 ± 3.0 months; bimodal 2.7 ± 3.7 months; bilateral 3.2 ± 7.9 months) As shown in Fig. 3, SSD patients with longer CC showed a significant difference of WRS at first fitting (CC^shorter^ 20.0 ± 28.9% vs. CC^longer^ 31.5 ± 24.7%; *p* = 0.04 Table 2) reaching similar values at 1-year post-implantation (*p* = 0.274). For bimodal hearing and bilateral implanted patients no significant difference was found (for all timepoints: *p* > 0.05 Table 2). No difference and no trend could be observed when analyzing patient groups regarding the received electrode arrays FLEX28, FLEXSOFT or STANDARD (data not shown). Same seemed for analyzing the first versus the second implanted ear (see Table 2).

**Table 2:** Audiometric result with regards to the cochlear coverage

	CC^shorter^				CC^longer^							Total							
	FF	1 M	3 M	12 M	FF	1 M		3 M		12 M		FF	Total	1 M	Total	3 M	Total	12 M	Total
SSD (n)	11	11	12	11	10	8		10		11		21		19		22		22	
preop PTA4 [dB ± SD]	100.7 ± 16.4			N/A ± N/A	103.3 ± 19.8	N/A ± N/A		N/A ± N/A		N/A ± N/A		100.9	± 17.9	N/A	± N/A	N/A	± N/A	N/A	± N/A
preop PTA^low^ [dB ± SD]	83.8 ± 28.0		± N/A	N/A ± N/A	101.3 ± 22.6	N/A ± N/A		N/A ± N/A		N/A ± N/A		92.9	± 26.4	N/A	± N/A	N/A	± N/A	N/A	± N/A
WRS at 65 dB [% ± SD]	20.0 ± 28.9	26.4 ± 26.9	35.0 ± 28.7	52.7 ± 28.6	31.5 ± 24.7	43.1 ± 13.9		45.5 ± 30.7		53.2 ± 27.5		25.5	± 26.9	33.4	± 23.5	39.8	± 29.4	52.9	± 27.4
*p*-values CC^shorter^ vs. CC^longer^	0.04*	0.097	0.144	0.274															
Numbers^65dB^ [% ± SD]	49.3 ± 39.9	83.6 ± 14.3	84.5 ± 20.2	96.4 ± 9.2	68.3 ± 30.4	68.2 ± 38.9		84.0 ± 30.9		89.2 ± 21.9		58.1	± 36.4	75.9	± 29.7	84.3	± 25.2	92.6	± 17.1
Numbers^50%^ [dB ± SD]	48.2 ± 5.8	42.6 ± 4.9	41.6 ± 6.6	38.1 ± 5.2	43.6 ± 5.1	42.5 ± 6.6		42.6 ± 6.8		43.9 ± 5.5		45.8	± 5.8	42.6	± 5.6	42.1	± 6.3	40.9	± 5.8
Bimodal (n)	10	15	16	15	20	26		31		35		30		31		47		50	
preop PTA4 [dB ± SD]	89.2 ± 16.5	N/A ± N/A	N/A ± N/A	N/A ± N/A	87.9 ± 13.4	N/A ± N/A		N/A ± N/A		N/A ± N/A		88.4	± 14.5	N/A	± N/A	N/A	± N/A	N/A	± N/A
preop PTA^low^ [dB ± SD]	80.0 ± 25.1	N/A ± N/A	N/A ± N/A	N/A ± N/A	70.1 ± 23.1	N/A ± N/A		N/A ± N/A		N/A ± N/A		73.9	± 24.2	N/A	± N/A	N/A	± N/A	N/A	± N/A
WRS at 65 dB [% ± SD]	13.0 ± 17.8	19.3 ± 17.3	27.5 ± 21.7	48.7 ± 21.4	10.5 ± 15.6	30.0 ± 27.4		33.7 ± 29.8		39.4 ± 26.6		11.3	± 16.1	26.1	± 24.5	31.6	± 27.2	42.2	± 25.3
*p*-values CC^shorter^ vs. CC^longer^	0.381	0.143	0.235	0.203															
Numbers^65dB^ [% ± SD]	48.0 ± 36.1	65.9 ± 27.4	77.5 ± 29.0	80.0 ± 36.3	40.9 ± 33.4	71.0 ± 30.3		79.3 ± 27.4		76.3 ± 32.9		43.3	± 34.1	69.1	± 29.1	78.6	± 27.7	77.4	± 33.7
Numbers^50%^ [dB ± SD]	45.2 ± 4.3	46.9 ± 5.4	43.5 ± 5.1	47.9 ± 5.8	47.9 ± 5.8	47.9 ± 8.0		48.3 ± 13.7		43.1 ± 5.5		47.3	± 5.5	47.4	± 6.8	46.4	± 11.2	44.4	± 5.8
Bilateral																			
First Ear (n)	4	3	4	4	5	3		5		4		9		6		9		8	
preop PTA4 [dB ± SD]	88.8 ± 30.4	N/A ± N/A	N/A ± N/A	N/A ± N/A	87.7 ± 16.9	N/A ± N/A		N/A ± N/A		N/A ± N/A		88.2	± 22.6	N/A	± N/A	N/A	± N/A	N/A	± N/A
preop PTA^low^ [dB ± SD]	88.7 ± 35.7	N/A ± N/A	N/A ± N/A	N/A ± N/A	63.3 ± 23.3	N/A ± N/A		N/A ± N/A		N/A ± N/A		74.8	± 30.9	N/A	± N/A	N/A	± N/A	N/A	± N/A
WRS at 65 dB [% ± SD]	25.0 ± 17.8	32.5 ± 45.9	50.0 ± 25.5	58.8 ± 23.9	17.5 ± 22.5	46.7 ± 33.3		35.0 ± 33.4		21.7 ± 22.5		21.3	± 19.2	41.0	± 33.8	42.5	± 28.7	42.9	± 29.1
Numbers^65dB^ [% ± SD]	65.0 ± 17.3	35.0 ± 40.4	90.0 ± 20.0	100.0 ± 0	58.0 ± 44.9	65.0 ± 45.1		71.7 ± 40.2		60.0 ± 56.6		61.1	± 33.7	50.0	± 42.8	79.0	± 33.5	84.0	± 35.8
Numbers^50%^ [dB ± SD]	45.7 ± 5.5	47.2 ± 9.5	45.0 ± N/A	N/A ± N/A	46.2 ± 6.9	51.6 ± 2.2		47.9 ± 8.6		N/A ± N/A		45.9	± 5.8	49.8	± 5.5	47.2	± 7.2	N/A	± N/A
Second Ear (n)	1	1	3	2	7	9		5		7		8		10		8		8	
preop PTA4 [dB ± SD]	80.9 ± 4.1	N/A ± N/A	N/A ± N/A	N/A ± N/A	98.3 ± 13.5	N/A ± N/A		N/A ± N/A		N/A ± N/A		93.9	± 14.0	N/A	± N/A	N/A	± N/A	N/A	± N/A
preop PTA^low^ [dB ± SD]	47.5 ± 25.9	N/A ± N/A	N/A ± N/A	N/A ± N/A	74.1 ± 32.0	N/A ± N/A		N/A ± N/A		N/A ± N/A		67.4	± 31.8	N/A	± N/A	N/A	± N/A	N/A	± N/A
WRS at 65 dB [% ± SD]	35.0 ± N/A	65.0 ± N/A	23.0 ± 23.1	27.5 ± 31.8	12.1 ± 18.2	30.6 ± 32.6		22.0 ± 29.3		36.4 ± 37.2		15.0	± 18.7	34.0	± 32.6	22.5	± 25.5	34.4	± 34.3
Numbers^65dB^ [% ± SD]	30.0 ± 51.9	100.0 ± N/A	100.0 ± N/A	80.0 ± N/A	63.8 ± 26.2	78.9 ± 34.4		76.7 ± 38.3		73.3 ± 38.8		54.5	± 35.6	81.0	± 33.1	80.0	± 36.1	74.3	± 35.5
Numbers^50%^ [dB ± SD]	49.2 ± 8.1	N/A ± N/A	N/A ± N/A	37.0 ± N/A	43.4 ± 2.8	42.8 ± 3.4		43.4 ± 7.0		39.8 ± 3.8		45.9	± 5.9	42.8	± 3.4	43.4	± 7.0	39.1	± 3.4
Both ears	5	4	7	6	12	12		10		11		17		16		17		16	
preop PTA4	73.2 ± 37.1	N/A ± N/A	N/A ± N/A	N/A ± N/A	72.1 ± 29.0	N/A ± N/A		N/A ± N/A		N/A ± N/A		92.4	± 19.0	N/A	± N/A	N/A	± N/A	N/A	± N/A
preop PTA^low^	85.9 ± 23.4	N/A ± N/A	N/A ± N/A	N/A ± N/A	95.7 ± 16.2	N/A ± N/A		N/A ± N/A		N/A ± N/A		73.0	± 31.8	N/A	± N/A	N/A	± N/A	N/A	± N/A
WRS at 65 dB [% ± SD]	27.0 ± 16.0	51.3 ± 28.7	38.6 ± 26.7	48.3 ± 28.4	15.8 ± 19.0	33.8 ± 32.6		43.1 ± 35.4		43.3 ± 39.7		19.1	± 18.5	38.1	± 31.7	34.4	± 28.4	41.2	± 32.9
*p*-values CC^shorter^ vs. CC^longer^	0.053	0.149	0.152	0.095															
Numbers^65dB^ [% ± SD]	45.0 ± 38.3	72.0 ± 42.1	92.0 ± 17.9	93.3 ± 11.5	62.9 ± 32.0	67.9 ± 40.0		76.2 ± 36.6		76.0 ± 37.2		58.6	± 33.5	68.9	± 39.4	80.6	± 32.8	80.0	± 33.4
Numbers^50%^ [dB ± SD]	47.4 ± 6.5	47.2 ± 9.5	45.0 ± N/A	37.0 ± N/A	44.8 ± 5.1	47.2 ± 5.4		45.3 ± 7.4		39.8 ± 3.8		45.9	± 5.7	47.2	± 5.8	45.3	± 6.9	39.1	± 3.4

Pure tone audiometric results and speech perception for the different groups single sided deafness (SSD), bimodal, and bilateral. For pure tone audiometry the pure tone average of the 4 frequencies 0.5, 1, 2, and 4 kHz (PTA4) and the PTA of the lower frequencies 0.125, 0.25, and 0.5 kHz (PTA^low^) were calculated. Regarding speech perception the word recognition score (WRS) at 65 dB, numbers at 65 dB (numbers^65dB^) in percent, and the sound pressure level for perception of 50% of the numbers (numbers^50%^) are given. The timepoints of first fitting (FF), 1 months (1 M), 3 months (3 M), and 12 months (12 M) are depicted. For all patient groups the values for those patients with a shorter cochlear coverage (CC^shorter^) and longer cochlear coverage (CC^longer^) are given. For subgroup analysis the median cochlear coverage was identified at 65% for SSD and bimodal hearing patients, at 70% for bilateral patients

*n* number of patients, *SD* standard deviation, *vs.* versus

*Significant* p*-values (*p* < 0.05)

Significant *p*-values (*p* < 0.05) are marked in bold with an asterisk

## Discussion

Data of this retrospective analysis of 131 CI patients suggests better speech perception for SSD-patients with deep insertion within the first year after implantation. For both, bimodal and bilateral patients, no significant difference between longer and shorter coverage was eminent. This observation highlights the importance of evaluating CI-patient categories separately. However, this data should be interpreted with caution, as various confounders apply to each specific group: (1) SSD: dependance on the implanted ear is not as significant as for other patient groups and therefore hearing effort might be perceived differently. In addition, SSD-patients had a short period of hearing loss with up to 2 years and underlying causes are often idiopathic sudden hearing loss [43], as in our cohort with more than 80%. Previous studies have shown beneficial effect on shorter duration of deafness upon implantation [44, 45]. (2) Bimodal patients: level of training, time of hearing aid usage and cause of hearing loss differ individually [43]. (3) Bilateral implanted patients: probably the most heterogeneous group of CI patients with both first and second implanted ear are often analyzed all together due to small sample sizes. Furthermore, in our cohort, patients seem to have longer CC (minimum around 60%). Further limitations of this study: (1) two categories of CI patients are not represented: asymmetric hearing and EAS. (2) The CDL measurements were performed with OTOPLAN version 3.0. (3) Patients in the SSD and bilateral group were fairly younger, than in the bilateral implanted group, which could be due to the cause of hearing loss (e.g. idiopathic sudden hearing loss in SSD patients). (4) This is a retrospective study, with limitations inherent to the characteristics of a retrospective study, such as small sample sizes, missing data with regards to different time points and duration of deafness. (5) Hence no postoperative CT scans with electrode array were available, no true insertion depth is depicted in this study. For the present analysis, the estimated CC-values of the preoperative CT-scan calculated by the OTOPLAN software were used. However, postoperative stenvers x-ray did suggest a full insertion and all patients with partial insertion were excluded from the analysis.

Strengths of the study is the analysis of the three different CI patient categories SSD, bimodal, and bilateral, separately. To date, only one further retrospective study on the analysis of bimodal patients exists, outlining an increased speech perception for deeper insertion [46]. All other study groups did not differentiate between patient categories, and therefore, this data should be interpreted with caution [30–35]. Most of the studies observed an increased speech perception with deeper insertion: A recent study of Weller et al. evaluated a large cohort of a total of 154 ears finding a linear correlation of increased speech perception with a deeper insertion and recommended an optimal CC of 79–82%. Confounder of this study is the heterogeneity of the cohort since data of bilaterally implanted and EAS stimulated patients were analyzed altogether. In addition, patients received a wide range of different lengths of electrodes (MED EL: Flex 20–20 mm, Flex 24–24 mm, Flex 26–26 mm, Flex 28–28 mm, Flex soft–31.5 mm) resulting in a wide range of CC [30]. The group around Alothman agrees with the hypothesis of increased speech perception with longer CC, which they showed in a study of 57 prelingually deafened children (85 ears) with uni- or bilateral implantation, who received either Flex 28 (28 mm) or Form 24 (24 mm) [32]. When comparing long-term speech perception (more than 4 years) between Flex 24 (24 mm) and Flex soft (31.5 mm) Canfarotta et al. found positive correlation with deeper insertion [47]. Focusing on the frequency-to-place mismatch in EAS and CI-alone patients, the same study group found a smaller deviation in patients with longer cochlear coverage, however accompanied with a certain degree of variability [34]. The same study group suggested in another study that the relationship between insertion depth and speech perception might depend on the array design (perimodiolar versus straight) which would highlight the importance of the auditory periphery in speech perception. However, it seems they have included different patient categories, which is not stated clearly enough to the reader [35]. Rossberg et al. also evaluated the influence of different electrode array designs (lateral wall versus perimodiolar) and found in perimodiolar a decreased and in straight electrode arrays an increased speech perception with longer cochlear coverage [31]. A study from Mlynski et al. did not find any correlation between insertion depth and speech perception not stating which kind of CI patient group they were investigating [21].

Comparing recent studies with the current study holds certain challenges, since some study groups were investigating other influencing factors like design of electrode array (straight versus lateral wall versus perimodiolar), included patients with significantly shorter electrode arrays (range of 22 mm up to 31.5 mm), analyzing results of all sorts of CI-patients (SSD, bimodal, bilateral, and EAS) all together, or focusing on frequency-to-place mismatch. In summary, developing speech perception is certainly influenced by a whole range of factors–of these, cochlear coverage is only one small factor within the grant equation. Nevertheless, the results of the current study hold value and support the hypothesis of deep insertion for better speech perception for SSD-patients. This patient group might be even the easiest to assess since the cause for hearing loss is often similar with a short time span between onset of hearing loss and receiving the implant. Certainly, bimodal and bilateral treated patients hold a large heterogeneity to these aspects, and even conducting prospective studies might not achieve to generate a homogeneous cohort at all. For prospects, cochlear implant registry trials could facilitate to evaluate comparable objectives.

## Conclusion

Deeper insertion seems to influence speech perception in SSD patients within the first year after implantation. Essentially, after the 12 month’s point, we found no difference in both groups with a cochlear coverage below and above the calculated median cutoff. However, these results should be interpreted with caution, since development of speech perception with CI is influenced by a whole range of factors. Thus, further investigations are required to create optimized analysis with larger subgroups and detailed evaluation including also longer electrodes for cochleae with very long cochlear duct length.

## Data Availability

Original data is available on demand.

## Code availability

Statistical analysis was performed using SPSS (version 29) software (SPSS Inc, Chicago, IL). Shapiro-Walk test was used to test for normative distribution. All figures were created with SPSS or Microsoft Excel version 1906. The measurement data would be made available upon request to the authors after a formal data-sharing agreement has been reached.

## Declarations

### Consolidated standard of reporting trial statement

Patient flow diagram see Fig. 1.
